# Extracellular DNA-induced antimicrobial peptide resistance in *Salmonella enterica* serovar Typhimurium

**DOI:** 10.1186/1471-2180-13-115

**Published:** 2013-05-24

**Authors:** Lori Johnson, Shawn R Horsman, Laetitia Charron-Mazenod, Amy L Turnbull, Heidi Mulcahy, Michael G Surette, Shawn Lewenza

**Affiliations:** 1 Department of Microbiology, Immunology and Infectious Diseases, University of Calgary, HRIC Building, Room 2C66, 3330 Hospital Drive NW, Calgary, AB, T2N 4N1, Canada; 2 Department of Medicine, Farncombe Family Digestive Health Research Institute, Health Sciences Centre, McMaster University, Rm 3N6-9, 1280 Main Street West, Hamilton, ON, L8S 4K1, Canada

**Keywords:** *Salmonella*, *phoPQ*, *pmrAB*, Antimicrobial peptides, Antibiotic resistance, Extracellular DNA, Biofilms, Immune evasion

## Abstract

**Background:**

The *Salmonella enterica* serovar Typhimurium PhoPQ two component system (TCS) is activated by low Mg^2+^ levels, low pH and by antimicrobial peptides (AP). Under Mg^2+^ limitation, the PhoPQ system induces *pmrD* expression, which post-translationally activates the PmrAB TCS. PhoPQ and PmrAB control many genes required for intracellular survival and pathogenesis. These include the polymyxin resistance (*pmr)* operon, which is required for aminoarabinose modification of LPS and protecting the outer membrane from antimicrobial peptide disruption and killing. Extracellular DNA is a ubiquitous polymer in the matrix of biofilms and accumulates in some infection sites. Extracellular DNA chelates cations and thus activates the *Pseudomonas aeruginosa* PhoPQ/PmrAB systems, leading to expression of the orthologous *arn* (*pmr*) operon.

**Results:**

Here we show that extracellular DNA induces expression of the *S*. Typhimurium *pmr* antimicrobial peptide resistance operon in a PhoPQ and PmrAB-dependent manner. Induction of the *pmr* genes by DNA was blocked when present with excess Mg^2+^. Exogenous DNA led to increased resistance of planktonic cultures to aminoglycosides, antimicrobial peptides (AP) and ciprofloxacin, but only AP resistance was PhoPQ/PmrAB-dependent. Extracellular DNA was shown to be a matrix component of *S*. Typhimurium biofilms cultivated in flow chambers and on glass surfaces. A *pmrH-gfp* fusion was highly expressed in flow chamber biofilms cultivated in medium with repressing levels of 10 mM Mg^2+^ and co-localized with eDNA. Expression of *pmrH-lux* was monitored in plastic peg biofilms and shown to require PhoPQ and PmrAB. Biofilms had higher levels of *pmrH* expression compared to planktonic cultures. We propose that DNA accumulation in biofilms contributes to the increased *pmrH-lux* expression in biofilms.

**Conclusions:**

The *Salmonella* PhoPQ/PmrAB systems and antimicrobial peptide resistance are activated by the cation chelating properties of extracellular DNA. DNA-induced AP resistance may allow immune evasion and increased survival of *S.* Typhimurium biofilms formed during extracellular growth stages of an infection or outside the host.

## Background

*Salmonella enterica* serovar Typhimurium is an enteroinvasive bacterial pathogen typically encountered by ingesting contaminated food or water. *S*. Typhimurium causes self-limiting gastroenteritis in humans and typhoid-like fever in mice [1,2]. Greater than 99% of the bacteria in murine salmonellosis are killed in the stomach or passed out of the gut [2], but *S*. Typhimurium that survive passage through the acidic stomach environment enter into the small intestine, where upon they transverse the intestinal epithelial barrier. The bacteria are then phagocytosed by macrophages or they can actively invade both phagocytic and non-phagocytic cells using a type III secretion system [1]. Following invasion, *Salmonella* disseminates throughout the body leading to a systemic typhoid-like infection [2].

S*almonella* forms biofilms on abiotic surfaces such as plastic and egg conveyer belts, which may have a role in environmental survival of this organism [3,4]. Biofilm formation and aggregation in *S. enterica* serovar Typhimurium is exemplified by the rdar colony morphology, where colonies grown on media containing Congo red are *r*ed, *d*ry, and *r*ough [5,6]. This morphology requires the production of curli fimbriae and multiple exopolysaccharides [7,8]. *S*. Typhimurium also grows enmeshed in EPS rich biofilms on the surface of gallstones, which may contribute to inefficient antibiotic treatment and facilitates typhoid carriage [9,10]. Biofilm shedding from colonized gallstones is likely a source of recurring infections [11].

The PhoPQ two-component system is important for intracellular survival within macrophages. Limiting Mg^2+^, low pH and the presence of antimicrobial peptides are PhoPQ-activating signals in culture [12,13] but low pH and antimicrobial peptides are important activating signals during intracellular macrophage growth [14]. The PmrAB two component system responds to Fe^3+^ and low pH, and is activated under Mg^2+^ limiting conditions by a post-translational mechanism involving PmrD, a PhoPQ-regulated protein. PmrD prevents the dephosphorylation of PmrA by PmrB, thus activating the expression of PmrA-regulated genes [15]. The *pmrHFIJKLM* operon is directly regulated by PmrAB, is induced during phagocytosis and is required for survival from host antimicrobial peptide production [16]. The *pmr* operon encodes an LPS modification system that is responsible for aminoarabinose modification of the lipid A moiety of LPS. Reducing the negative charge of the bacterial surface with aminoarabinose is critical for reducing the membrane damaging effects of cationic antimicrobial peptides.

We recently demonstrated that DNA is a cation chelator that induces expression of the *Pseudomonas aeruginosa arnBCADTEF-ugd* (*PA3552-PA3559*; *pmr*) operon in DNA-enriched planktonic cultures and biofilms [17]. DNA sequesters cations and creates a condition that resembles a Mg^2+^-limited environment, similar to known chelators like EDTA. Expression of this operon was required for very high levels of biofilm resistance to antimicrobial peptides and partially contributed to aminoglycoside resistance [17]. During Mg^2+^ limiting growth conditions, the *P. aeruginosa* PhoPQ and PmrAB systems are both required for expression of the *arn* operon [18,19]. Both the PhoPQ and PmrAB systems respond to Mg^2+^ limitation in *P. aeruginosa*, and there is no PmrD ortholog to connect the two pathways. In addition, the *P. aeruginosa* PhoQ sensor does not directly detect antimicrobial peptides, and the PmrB sensor does not respond to trivalent metals [18]. Extracellular DNA also induces the expression of PmrAB-regulated spermidine synthesis genes, which results in the production of the polycation spermidine on the surface and protection of the outer membrane from antimicrobial peptide treatment [20]. Both the *arn* and spermidine synthesis (*PA4773-PA4775)* clusters were induced in biofilms formed by a *bfmR* mutant of *P. aeruginosa* that accumulated more eDNA than wild-type biofilms [21]. When sufficient DNA accumulates in *P. aeruginosa* biofilms, or in the cystic fibrosis (CF) lung where the concentration of DNA is very high and leads to viscous sputum production in CF patients [22,23], the expression of these DNA-induced surface modifications likely protect from host antimicrobial peptide killing. Therefore, we wanted to determine if extracellular DNA plays a general role in antimicrobial peptide resistance by imposing a cation limitation on *S*. Typhimurium biofilms and activating the PhoPQ/PmrAB systems, similar to *P. aeruginosa*.

## Results and discussion

### Extracellular DNA induces expression of the *Salmonella pmr* operon

**Figure 1:**
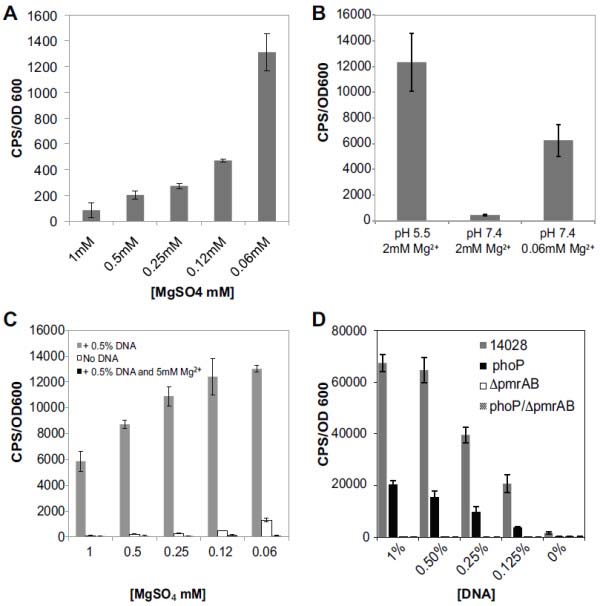
**Cation chelation by extracellular DNA induces expression of the *pmr *operon.** (**A**) Expression of *pmrH-lux* in NM2 media (pH7.4) in final Mg^2+^ concentrations ranging from 1 mM to 0.06 mM. (**B**) Expression of *pmrH-lux* in NM2 media pH5.5 and pH7.4, in varying Mg^2+^ concentrations. (**C**) Expression of *pmrH-lux* in NM2 media (pH7.4) in varying Mg^2+^ concentrations ranging from 1 mM to 0.06 mM (white bars), media supplemented with 0.5% DNA (5 mg/ml) (grey bars) or 0.5% DNA plus excess 5 mM Mg^2+^ (black bars). (**D**) Expression of *pmrH-lux* in NM2 media (pH7.4) containing repressing levels of Mg^2+^ (1 mM) and supplemented with increasing concentrations of extracellular DNA, as indicated. Expression was measured in strains 14028, *phoP*, *pmrAB* and *phoP/pmrAB* mutants. In all experiments, gene expression was measured every 20 minutes for 18 hours and the maximal gene expression (t = ~7 hrs) is shown. The values shown are the means from experiments done in triplicate and the error bars represent the standard deviation.

A low copy, plasmid-encoded transcriptional *lux* fusion to the *pmrH* promoter [24] was expressed in *Salmonella enterica* serovar Typhimurium 14208 under various planktonic growth conditions. At pH 7.4, the *pmrH-lux* reporter was repressed at 1 mM Mg^2+^ but was induced 13-fold in a stepwise fashion as the Mg^2+^ concentration was decreased to 0.06 mM (Figure 1A). The *pmrH-lux* fusion was most highly expressed under low pH (5.5) (Figure 1B), even with the addition of up to 50 mM Mg^2+^ (data not shown). These observations were consistent with previous reports of *pmr* expression conditions [12,13]. A fixed concentration of DNA (0.5%, 5 mg/ml) was added to cultures grown in a range of Mg^2+^ concentrations between 1 mM and 0.06 mM. In each Mg^2+^ concentration, the addition of 0.5% DNA caused a strong induction of *pmrH-lux* expression (up to 70-fold) (Figure 1C). To confirm that DNA induced *pmrH-lux* expression via cation chelation, we added exogenous 5 mM Mg^2+^, which was sufficient to prevent DNA-mediated induction of *pmrH-lux* (Figure 1C). Taken together, these observations indicate that DNA chelates and sequesters Mg^2+^ and the cation chelating activity can be blocked with excess Mg^2+^.

Next, we monitored *pmrH-lux* expression in wild type, *phoPQ*, *ΔpmrAB* and *phoPQ/ΔpmrAB* mutant backgrounds. DNA-induced expression did not occur in *ΔpmrAB* or *phoPQ/ΔpmrAB* double mutants, indicating an absolute requirement for *pmrAB* in responding to extracellular DNA (Figure 1D). A *phoPQ* mutant was still able to partially respond to extracellular DNA, which was likely due to the presence of PmrAB (Figure 1D). In summary, extracellular DNA imposes a cation limitation on *S.* Typhimurium, leading to induction of the *pmrH* promoter in a PhoP and PmrA-dependent manner.

### Extracellular DNA is a matrix component *S.* Typhimurium biofilms

**Figure 2:**
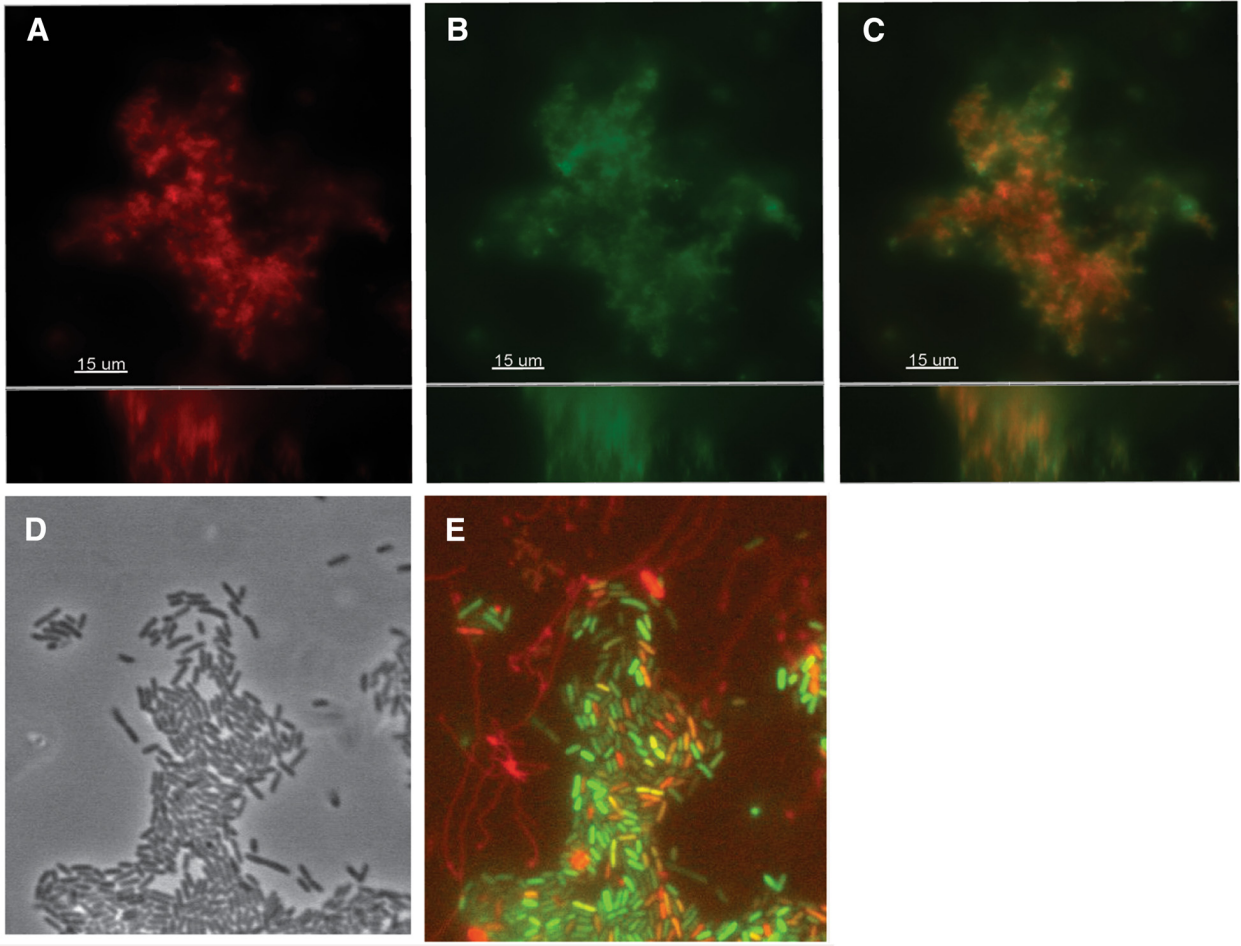
**Extracellular DNA accumulates in the matrix of *S. *Typhimurium biofilms.** Biofilms of strain 14028 were cultivated in flow chambers at 37°C for 2 days in LB medium and stained for extracellular DNA. Cells in the biofilm were stained with the membrane staining dye FM 4–64 (**A**). The middle panel depicts the accumulation of extracellular DNA with TOTO-1 staining (**B**). The images are merged on the right (**C**). The large image shows the xy plane and the bottom panel shows the xz plane. The scale bar equals 15 μM. The wild-type 14028 strain carrying the *pmrH-gfp* construct forms aggregates on the surface of glass (**D**). The merged image of green fluorescence from *pmr* expression and red from propidium iodide staining, which stains both dead cells and extracellular DNA (**E**).

While radar colony biofilms and biofilms on gallstones produce an extracellular matrix composed of multiple EPS species, the presence of extracellular DNA has not been well reported [5,6]. Here we cultivated flow chamber biofilms of *S. enterica* serovar Typhimurium at 37°C for 48 hours. To determine if DNA accumulates in the matrix of *S.* Typhimurium biofilms, we stained for the presence of extracellular DNA with Toto-1. Large aggregates formed within 2 days that were 20–30 μM in height and stained positive for extracellular DNA (Figure 2A-C), illustrating that eDNA accumulates in *Salmonella* flow chamber biofilms. Biofilms were also cultivated on glass cover slips immersed in growth media and stained with propidium iodide, which showed the accumulation of eDNA fibers extending from an aggregate (Figure 2D,E).

### DNA-enriched planktonic cultures show increased antibiotic resistance

The presence of extracellular DNA may lead to increased *S.* Typhimurium *pmr* expression, increased AP resistance and thus help to explain the antibiotic resistance phenotype that is characteristic of biofilms. To determine the influence of DNA on antibiotic resistance, we tested the antibiotic susceptibility of *S.* Typhimurium 14028 planktonic cultures in the presence and absence of exogenous DNA (pH 7.4). The addition of 0.5% DNA (5 mg/ml) led to a 16-fold increased resistance to polymyxin B and colistin, a 4-fold increased resistance to gentamicin and a >4 fold increase in resistance to ciprofloxacin (Table 1). Both *phoPQ* and *pmrAB* mutants did not demonstrate DNA-induced resistance to polymyxin B and colistin. However, both mutants had parental levels of DNA-induced resistance to gentamicin and ciprofloxacin, indicating that resistance to these antibiotics was independent of the *phoPQ* and *pmrAB* systems (Table 1). Extracellular DNA is known to bind to aminoglycosides through electrostatic interactions [25], and it was recently shown that exogenous DNA shields *P. aeruginosa* from aminoglycoside killing, independent of the *pmr* resistance mechanism [26].

**Table 1:** **Extracellular DNA induces antibiotic resistance in *S. *** **Typhimurium**

**Strain**	**Minimal inhibitory concentration (MIC)**							
	**Polymyxin B**		**Colistin**		**Gentamicin**		**Ciprofloxacin**	
	**-**	**+ DNA**^**a**^	**-**	**+ DNA**^**a**^	**-**	**+ DNA**^**a**^	**-**	**+ DNA**^**a**^
14028	1	16	1	16	0.125	0.5	0.125	>0.5
*phoPQ*	1	0.5	1	1	0.125	0.25	0.125	>0.5
Δ*pmrAB*	0.5	0.5	0.5	0.5	0.125	0.5	0.125	>0.5

^**a**^ The minimal inhibitory concentration (MIC) values were determined in NM2 medium containing 1 mM Mg^2+^ (pH 7.4) with or without the addition of 0.5% fish sperm DNA-sodium salt (5 mg/ml).

The observation that *phoPQ* and *pmrAB* mutants showed an increased susceptibility to colistin and polymyxin B, in the presence of eDNA, indicated a role for PhoPQ/PmrAB-regulated phenotypes in resistance to membrane acting antimicrobial peptides, likely through the aminoarabinose modification of LPS via the *pmr* operon.

### The *pmr* operon is highly expressed in biofilms

**Figure 3:**
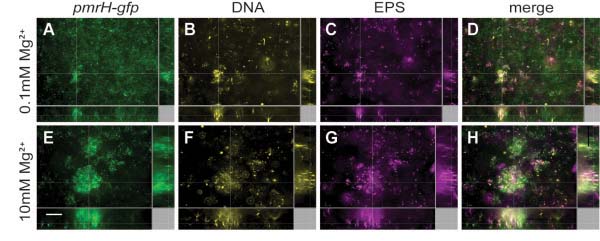
**The *pmrH-gfp *fusion is expressed in flow chamber biofilms under repressive high Mg**^**2+ **^**conditions.** Biofilms of strain 14028 expressing a plasmid-encoded *pmrH-gfp* construct were cultivated for 2 days in NM2 (pH 7.4) under inducing conditions with 0.1 mM Mg^2+^ (**A**-**D**) or under inducing conditions with 0.1 mM Mg^2+^ for 28 hours followed by the injection of excess 10 mM Mg^2+^ into the flow chambers for an additional 16 hours (**E**-**H**). Gfp fluorescence was monitored in **A**,**E**; extracellular DNA was stained in **B**,**F** (pseudocoloured yellow); EPS was stained in **C**,**G** (pseudocoloured purple); and the merged image of the three channels is shown in **D**,**H**. The scale bar equals 20 μM.

We wanted to determine if *pmrH* is expressed in biofilms due to the natural accumulation of eDNA released from lysed cells. Flow chamber biofilms were cultivated and monitored for the expression of a *pmrH-gfp* transcriptional fusion. As a positive control, biofilms were cultivated in NM2 containing 0.1 mM Mg^2+^, which we previously had shown was an inducing condition (Figure 1A). As expected, *pmrH-gfp* was expressed throughout the biofilm, which also stained positively for extracellular DNA with a second DNA stain Sytox Red, and stained positively for calcofluor white, which binds cellulose and other exopolysaccharides with β-1,4 linkages (Figure 3). We next cultivated biofilms in NM2 containing 0.1 mM Mg^2+^ for 28 hours and then introduced an extra 10 mM Mg^2+^ into the media for the next 16 hours of biofilm cultivation. We expected the exogenous addition of 10 mM Mg^2+^ to repress *pmrH* expression since 5 mM Mg^2+^ could completely repress expression in planktonic cultures in the presence of exogenous DNA (0.5%). However, *pmrH-gfp* was strongly expressed in biofilms grown in media despite repressing levels of Mg^2+^ (Figure 3). Extracellular DNA was visualized in large microcolonies with Sytox Red staining and appeared to generally colocalize with *pmrH-gfp* expression. This observation suggests that the exogenous addition of excess Mg^2+^ to pre-formed biofilms could not gain access or was not in sufficient concentration to neutralize the cation chelating properties of endogenous matrix eDNA. Alternatively, the long half-life of Gfp may also contribute to the fluorescence signal detected after 46 hours of growth.

**Figure 4:**
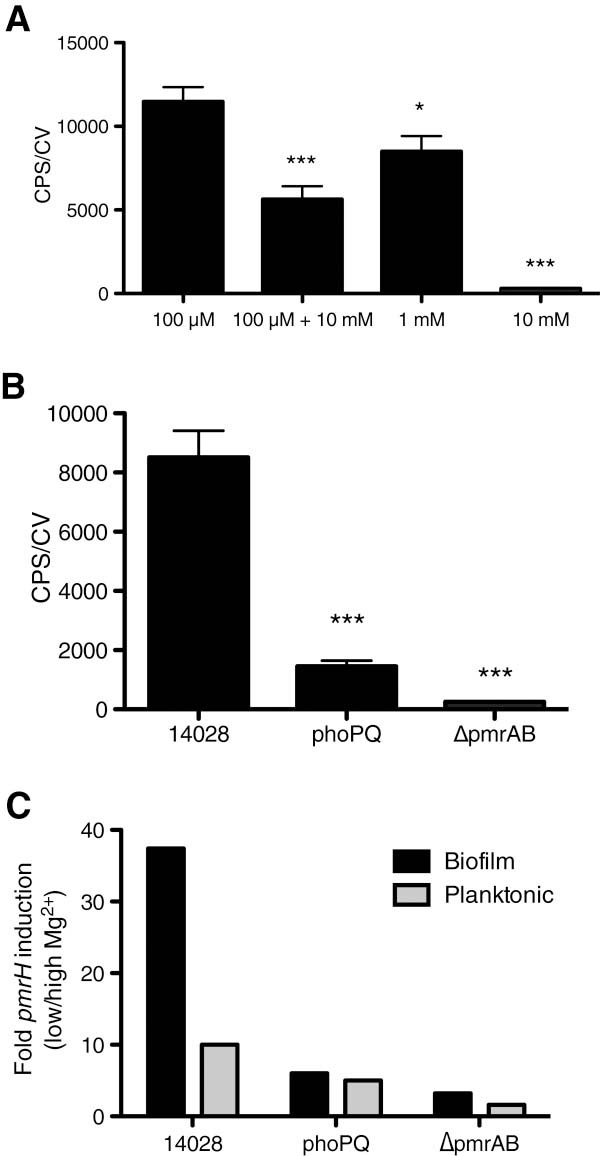
**Expression of *pmrH-lux *in peg-adhered biofilms requires PhoPQ and PmrAB.** (**A**) Gene expression was measured in plastic peg-adhered biofilms cultivated 18 hrs in NM2 media with 100 μM Mg^2+^, 100 μM Mg^2+^ then spiked with 10 mM Mg^2+^ for 4 hrs, 1 mM Mg^2+^ or 10 mM Mg^2+^. Values shown are the average of 8 replicates with the standard deviation of gene expression (CPS) normalized to biofilm biomass (CV). Values that differ significantly from the controls (100 μM Mg^2^) are marked with an asterisk (*, p < 0.05; ***, p < 0.001 by unpaired *t* test). (**B**) Under repressing levels of 1 mM Mg^2+^, *pmrH-lux* expression was measured in biofilms formed by 14028, *phoPQ* and Δ*pmrAB* strains. Values shown are the average of 8 replicates with the standard deviation of gene expression (CPS) normalized to biofilm biomass (CV). Values that differ significantly from the controls (14028) are marked with an asterisk (***, p < 0.001 by unpaired *t* test). (**C**) The normalized *pmrH* gene expression under inducing conditions (100 μM Mg^2+^) was divided by the normalized *pmrH* expression in repressing conditions (10 mM Mg^2+^) and shown as a fold induction value from either peg-adhered biofilms (black bars) or planktonic cultures (grey bars). Each experiment was repeated three times.

To overcome the potential issue with stable Gfp reporters, we measured gene expression in 96-well format peg-adhered biofilms using the *pmrH-lux* reporter. In Figure 4A, biofilms cultivated in limiting Mg^2+^ (100 μM) showed the highest expression levels, and expression decreased if biofilms were cultivated in excess Mg^2+^ conditions (1–10 mM). Biofilms that were cultivated overnight in limiting Mg^2+^ conditions but were treated with 10 mM Mg^2+^ for 4 hours, showed a partial repression (Figure 4). This result confirms that the residual *pmrH-gfp* expression after the addition of excess Mg^2+^ for 16 hours (Figure 3) is due to stable *gfp* expression, as the *pmrH-lux* reporter was responsive to the addition of excess Mg^2+^.

We measured *pmrH-lux* expression in conditions with repressing levels of Mg^2+^ (1 mM), and showed that *pmrH* expression was dependent on both PhoPQ and PmrAB in biofilms (Figure 4B). Lastly, we calculated the fold induction values of *pmrH* between inducing (100 μM) and repressing Mg^2+^ levels (10 mM), simultaneously for both peg-adhered biofilms and the planktonic cultures that served as the inoculum for the biofilms. Interestingly, *pmrH* was more highly expressed in biofilms when compared to planktonic cultures (27-fold higher), and expression under all conditions required PhoPQ and PmrAB (Figure 4C). We propose that the higher *pmrH* expression levels in biofilms may be due to the accumulation of eDNA, which increases *pmrH* expression in biofilms but not planktonic cultures.

## Conclusion

We showed evidence that extracellular DNA is a component of the *S.* Typhimurium extracellular matrix when grown in biofilms. When added to planktonic cultures, eDNA chelates cations resulting in a Mg^2+^ limited environment and increased expression of the *pmr* operon. The *pmr* operon was more highly expressed in biofilms, when compared to planktonic cultures. Expression of *pmr* in biofilms and DNA-induced expression in planktonic conditions is dependent on the PhoPQ/PmrAB systems. The addition of eDNA to planktonic cultures also led to increased antimicrobial peptide resistance in a PhoPQ/PmrAB-dependent manner. Combined with our previous observations of DNA-induced antibiotic resistance mechanisms in *P. aeruginosa*[17], we propose that extracellular DNA has a general role as a cation chelator that induces antimicrobial peptide resistance in biofilms. DNA-induced resistance to antibiotics and antimicrobial peptides from the innate immune system may promote long-term survival of *S.* Typhimurium biofilms in the environment, on the surface of gallstones, or possibly in the extracellular phases of growth during intestinal infection.

## Methods

### Bacterial strains and growth conditions

*S. enterica* serovar Typhimurium strain ATCC 14028 was used as the reference strain in this study. The *phoPQ::Tn10-Tc*^*R*^ mutant was previously described [27], *ΔpmrAB::cat* was constructed as previously described [28], and the *phoPQ ΔpmrAB* mutant strain was constructed by P22-mediated transduction [29] of both mutations into the same background. Cultures were routinely grown overnight at 37°C with agitation in Luria Broth base (LB) supplemented with 50 μg/ml kanamycin, if necessary. Gene expression experiments were performed in NM2 defined minimal media with either high (7.4) or low (5.5) pH. NM2 growth medium includes the following components: 5 mM potassium chloride, 7.5 mM ammonium sulfate, 0.5 mM potassium sulfate, 1 mM monopotassium phosphate, 38 mM glycerol, 0.1% casamino acids, and 100 mM Tris (pH 7.4 or 5.5), supplemented with magnesium sulfate when indicated. When added, the source of extracellular DNA was fish sperm DNA-sodium salt (MJS BioLynx).

### Gene expression assays in planktonic cultures

Gene expression was performed in high throughput format using 96-well microplates as previously describe [17]. Briefly, overnight cultures were grown in LB supplemented with 50 μg/ml kanamycin as required, diluted 1/1000 into 150 μl of NM2 defined culture medium with MgSO_4_, DNA or both, in 96-well black plates with a transparent bottom (9520 Costar; Corning Inc.) and overlaid with 50 μl of mineral oil to prevent evaporation. Microplate planktonic cultures were incubated at 37°C in a Wallac Victor^3^ luminescence plate reader (Perkin-Elmer) and optical density (growth, OD_600_) and luminescence (gene expression, counts per second (CPS)) readings were taken every 20 minutes throughout growth.

### Biofilm and gene expression assays on pegs

Biofilms were cultivated on 96-well format, polystyrene pegs (Nunc-TSP) that were immersed in 150 μl of NM2 growth medium, as previously described [17]. After biofilm cultivation, non-adherent cells were removed by rinsing the pegs once in 20 mM Tris buffer (pH 7.4). Gene expression (CPS) from peg-adhered biofilms was measured by luminescence readings in the Wallac MicroBeta Trilux multi-detector (Perkin-Elmer). Biofilm formation on the pegs was quantitated by crystal violet (CV) staining as previously described [17]. Gene expression (CPS) on pegs was divided by the biofilm biomass (CV) to normalize gene expression to cell number (CPS/CV), and gene expression in planktonic culture was divided by the OD_600_ value of cells in suspension to normalize for cell number (CPS/OD_600_). Biofilms were cultivated in NM2 with limiting Mg^2+^ (100 μM) or high levels of Mg^2+^ (1–10 mM).

### Minimal inhibitory concentration (MIC) assay

The MIC values were determined using the broth microdilution procedure in 96-well microplates. Briefly, all strains were grown overnight in LB medium, sub-cultured into NM2 medium (1 mM Mg^2+^) (1/100 dilution) and grown to mid-log phase. All cultures were normalized to a common OD_600_ value and 10 μl of mid-log culture (~6 × 10^5^ cfu) was inoculated into 90 μl of NM2 media containing repressing levels Mg^2+^ (1 mM), with or without 5 mg/ml DNA-sodium salt. Microtitre plates containing the antibiotic dilution series and bacteria were incubated for 18 hours at 37°C. The MIC was determined as the concentration of antibiotic that reduced growth to an OD_600_ value less than 0.1. The median MIC values from three experiments are shown.

### Flow chamber biofilm cultivation and imaging

Biofilms were grown in flow chambers with channel dimensions of 1 × 4 × 40 mm as previously described but with minor modifications [30]. Autoclaved silicone tubing (VWR, .062 ID x .125 OD x .032 wall) was assembled and sterilized by pumping 0.5% hypochlorite solution through the flow chamber for 2 hours. For rinsing, sterile water was pumped though for 30 minutes followed by LB media for 30 minutes. Flow chambers were inoculated by injecting with a syringe, 400 μl of mid-log culture diluted to an OD_600_ of 0.02. After inoculation, chambers were left without flow for two hours to allow the bacteria to adhere, after which media was pumped though the system at a constant rate of 0.75 rpm (3.6 ml/hour). Biofilms were cultivated for 48 hours at 37°C in LB medium and stained with the membrane staining dye FM 4–64 (Invitrogen), the extracellular DNA stains TOTO-1 or Sytox Red (Invitrogen), or an EPS stain fluorescent brightener 28 (Sigma). Biofilms were imaged using a Leica DMI 4000 B widefield fluorescence microscope equipped with filter sets for blue (Ex 390/40, Em 455/50), green (Ex 490/20, Em 525/36) and red (Ex 555/25, Em 605/52) fluorescence using the Quorum Angstrom Optigrid (MetaMorph) acquisition software. Images were obtained with a 63 × 1.4 objective. Deconvolution was performed with Huygens Essential (Scientific Volume Imaging B.V.) and 3D reconstructions were generated using the Imaris software package (Bitplane AG).

### Monitoring *pmrH-gfp* expression in flow chamber biofilms

The promoter of *pmrH* was amplified from genomic DNA of *S. typhimurium* 14028 using the primer pair pmrF-1 (AGTCCTCGAGACTACCGGATGCTGCTTC) and pmrF-2 (AGTCGGATCCATTGCCAGTTAGCCGACA), digested with BamHI-XhoI and cloned into BamHI-XhoI-digested pCS21 upstream of a *gfpmut3* reporter [31]. The *pmrH-gfp* vector was moved into *S.* Typhimurium 14028 by electroporation. Flow chamber biofilms were cultivated in NM2 containing 0.1 mM Mg^2+^ for 28 hours and then 10 mM Mg^2+^ was introduced into the growth media for an additional 16 hours of biofilm cultivation prior to imaging.
